# The Immunological Impact of IL-1 Family Cytokines on the Epidermal Barrier

**DOI:** 10.3389/fimmu.2021.808012

**Published:** 2021-12-23

**Authors:** Tom Macleod, Anna Berekmeri, Charlie Bridgewood, Martin Stacey, Dennis McGonagle, Miriam Wittmann

**Affiliations:** ^1^ School of Molecular and Cellular Biology, University of Leeds, Leeds, United Kingdom; ^2^ Leeds Institute of Rheumatic and Musculoskeletal Medicine (LIRMM), University of Leeds, Leeds, United Kingdom; ^3^ National Institute for Health Research (NIHR) Leeds Biomedical Research Centre (BRC), The Leeds Teaching Hospitals, Leeds, United Kingdom

**Keywords:** IL-36 cytokine family, IL-1 family cytokines, IL-33, proteolytic regulation, IL-1alpha, IL-1beta, pathogen/commensal discrimination, IL-18

## Abstract

The skin barrier would not function without IL-1 family members, but their physiological role in the immunological aspects of skin barrier function are often overlooked. This review summarises the role of IL-1 family cytokines (IL-1α, IL-1β, IL-1Ra, IL-18, IL-33, IL-36α, IL-36β, IL-36γ, IL-36Ra, IL-37 and IL-38) in the skin. We focus on novel aspects of their interaction with commensals and pathogens, the important impact of proteases on cytokine activity, on healing responses and inflammation limiting mechanisms. We discuss IL-1 family cytokines in the context of *IL-4/IL-13* and *IL-23/IL-17* axis-driven diseases and highlight consequences of human loss/gain of function mutations in activating or inhibitory pathway molecules. This review highlights recent findings that emphasize the importance of IL-1 family cytokines in both physiological and pathological cutaneous inflammation and emergent translational therapeutics that are helping further elucidate these cytokines.

## Introduction

The IL-1 family is a large group of cytokines, all of which are expressed in human epidermis, and have well established roles in skin immunopathology. Collectively these cardinal immunological functions encompass anti-microbial host protection and barrier function maintenance or restoration *via* inflammation and inflammation resolution mechanisms. In addition to established pathological roles this family of cytokines are vital for maintaining the skin’s immunological barrier and wound repair, yet this physiological role often gets overlooked. This review aims to give an overview of the role IL-1 family proteins play in skin, focusing on novel findings in their physiological role, and provides context for a deeper understanding of pathway derangement with resultant cutaneous disease.

## The IL-1 Family Cytokines

**Figure 1:**
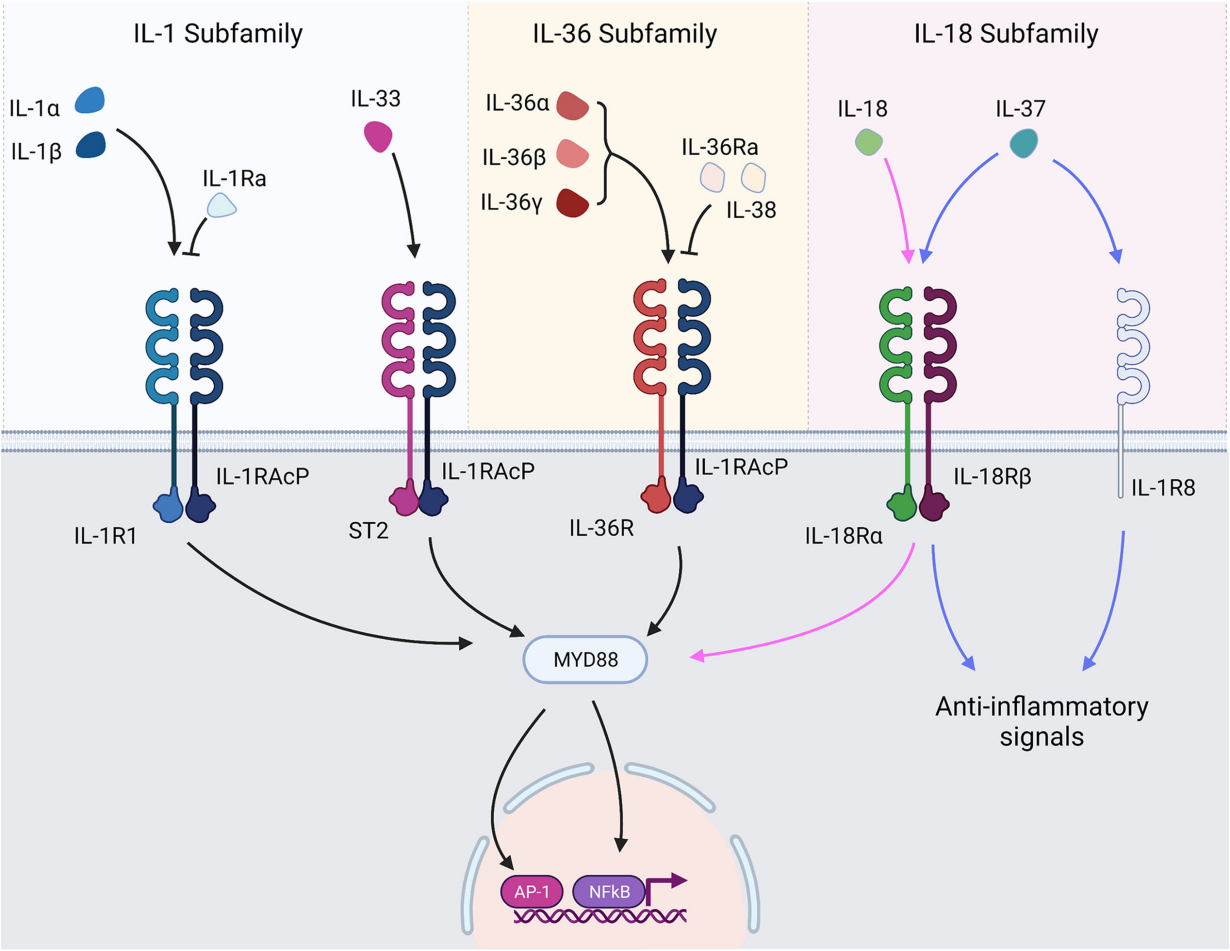
IL-1 superfamily cytokines and receptors. IL-1 family cytokines bind their respective receptors and transduce signals through TIR/MyD88 signalling pathways. The major signalling pathways result in activation of NF-κB and AP-1 and subsequent transcription of genes under their control. Receptor antagonists bind their respective receptors but prevent the recruitment of the accessory protein, thereby preventing signal transduction. IL-37 exerts anti-inflammatory signals following interaction with IL-1R8 and IL-18Rα.

The IL-1 superfamily (**Figure 1**) can be split into three smaller subfamilies according to structural similarities amongst the cytokine IL-1 consensus sequence and their primary ligand binding receptors: the IL-1, IL-18 and IL-36 subfamilies (1). The IL-1 subfamily has three agonists (IL-1β, IL-1α and IL-33) and a receptor antagonist (IL-1Ra). The IL-18 subfamily contains IL-18 as well as the anti-inflammatory cytokine IL-37 and IL-18 binding protein (IL-18BP). The IL-36 subfamily consists of three agonists – IL-36α, IL-36β and IL-36γ – and the antagonist IL-36Ra. It also contains the cytokine IL-38 whose function is not wholly understood yet is considered predominantly anti-inflammatory.

With the exception of IL-1Ra, all IL-1 family cytokines lack an N-terminal signal peptide and are therefore not secreted in a classical manner (2). Each cytokine is produced as an immature precursor that undergoes maturation by proteolytic truncation, the specificity of which differs from cytokine to cytokine. The activity of IL-1 family cytokines is hugely affected by this proteolytic maturation. Some cytokines such as IL-1α and IL-33 have biological activity in their immature form, yet others such as IL-1β and the IL-36 cytokines must undergo maturation to become active (1–3). Within the proteolytic maturation itself there exists more levels of regulation as variation in the specific truncations generated can affect the biological activity of the resulting mature cytokine. Therefore, changes to the proteolytic environment to which the IL-1 family cytokines are exposed can significantly affect the resulting inflammatory outcome and their subsequent impact on skin.

Signalling within the IL-1 family is a highly conserved process, with agonists binding their cognate receptors causing signal transduction *via* Toll/Interleukin-1 Receptor (TIR) and MyD88-dependent signalling pathways culminating in the activation of NF-κB and MAPK and subsequent NF-κB and AP-1 dependent gene transcription (4). As a result, many IL-1 family members generate similar pro-inflammatory responses. Nuances in cellular and tissue compartment expression, activation of IL-1 family cytokines and the responsive cell types, however, means IL-1 family cytokines can generate a huge variation of cellular responses and impart a broad impact on the immune system. The IL-1 family cytokines signal through IL-1 family receptors, some of which are shared amongst cytokines (**Figure 1**). IL-1 receptor biology is a complex topic in itself that is beyond the remit of this review and has been extensively reviewed elsewhere (5). However, to provide a contextual overview, all IL-1 family cytokines transduce signals in a similar manner. Agonists bind their cognate receptor, which subsequently recruits a membrane-bound accessory protein. Interaction between the ligand-bound receptor and the recruited accessory protein allows a signal to be transduced to the cell (6). As outlined in greater detail by **Figure 1**, some of the receptors are shared amongst IL-1 family cytokines. IL-1α, and IL-1β bind IL-1R1, IL-33 binds ST2, IL-18 and IL-37 bind IL-18Ra, IL-37 also binds a decoy receptor IL-1R8 through which it mediates some anti-inflammatory activity, and the IL-36 cytokines all bind IL-36R.

All IL-1 family cytokines are expressed, with some variation, within the skin. IL-1α is constitutively expressed by keratinocytes, yet is retained as intracellular stores (7, 8). IL-1β has a more specialised expression profile across the body, however in the skin most cell types present can express the cytokine, notably keratinocytes, fibroblasts and both resident and infiltrating myeloid cells (9, 10). Unlike IL-1α, IL-1β is only expressed following cell activation. IL-33 is only weakly expressed by keratinocytes in healthy human skin, but is strongly induced in the epidermis of inflamed skin (11). IL-18 is also expressed throughout the epidermis. Similarly to IL-1α, IL-18 is constitutively expressed by keratinocytes and retained within cells as intracellular stores, yet is only cleaved into its mature form following cell activation in the same manner as IL-1β (12). IL-37 exists in 5 isoforms termed IL-37a-e as a result of alternative splicing. The largest and most common isoform, IL-37b, is expressed by keratinocytes, monocytes and other immune cells within skin (13). Of the IL-36 cytokines, IL-36γ is the most strongly expressed in skin. Tape strip analysis of human skin show basal levels of IL-36γ are present in the outer layers of the epidermis, even in healthy skin (14). IL-36γ is constitutively expressed at low levels by keratinocytes, and strongly upregulated following activation (15, 16). The IL-36 cytokines are not constitutively expressed by other cell types present in skin but are inducible in myeloid cells following activation (17). In addition to functioning as cytokines, IL-1α, IL-33 and some isoforms of IL-37 are known to translocate to the nucleus where they can regulate gene expression through their DNA-binding capabilities (8, 13, 18).

## Sensing Environmental Threats

A number of IL-1 family proteins have a significant role in detecting environmental threats encountered in skin. Many have been added to the group of damage-associated molecular patterns (DAMPs) or “alarmins”.

### Protease Sensors

Several factors in IL-1 family member biology and expression characteristics make these cytokines excellent sensors of protease activity, alerting the immune system to potential threats. Although IL-1α, IL-33 and IL-37 exhibit a reduced level of activity in their immature form, IL-1β, IL-18, and the IL-36 cytokines require N-terminal truncation in order to engage their respective receptors (19). Proteolytic activation of IL-1 family cytokines is a tightly controlled process in order to license inflammation only when necessary. IL-1β and IL-18 activity is controlled by regulation of caspase-1, which itself remains inactive until inflammasome assembly occurs (20). The IL-36 agonists require precise cleavage to achieve maturity; truncation a single amino acid upstream or downstream of the correct site will generate an inactive cytokine (2). Indeed, as will be discussed later, a breakdown in the regulation of IL-1 proteolysis can lead to significant inflammatory disease.

Nevertheless, despite strict endogenous controls on the activity of IL-1 family cytokines, several have been shown to be activated by exogenous pathogen-derived and environmental proteases. IL-1β and IL-36γ are both processed to their active forms by the protease SpeB, secreted by *Streptococcus pyogenes* during infection (15). IL-36γ is also processed to its active form by Alp1, secreted by *Aspergillus fumigatus*, and both IL-36γ and IL-36α have been shown to be activated by *Staphylococcus aureus* and *Trichophyton rubrum* culture filtrates (15). Active IL-1β and IL-36 cytokines promote the development of Type I and Type III immune responses, which will facilitate clearance of bacterial and fungal pathogens. As such, the requirement of IL-1 family cytokines to undergo proteolytic cleavage to reach biological maturity also enables the cytokines to function as sensors of exogenous proteases and signal the presence of a threat.

### Alarmin Function

IL-1α has long been associated with an alarmin role owing to its constitutive expression maintaining an intracellular store of the cytokine, and its ability to bind its receptor and transduce signals in its immature form. In the event of cellular damage or programmed cell death IL-1α is released into the extracellular space and able to warn surrounding cells of damage or infection (21). Furthermore, the skin barrier is subjected to mechanical stressing as a physiological phenomenon and this too regulates the release of IL-1α (22). Whilst IL-1α has been long established in this role, recent work supports the case that other IL-1 family members may also function in an alarmin capacity despite not wholly fitting the classical definition.

**Figure 2:**
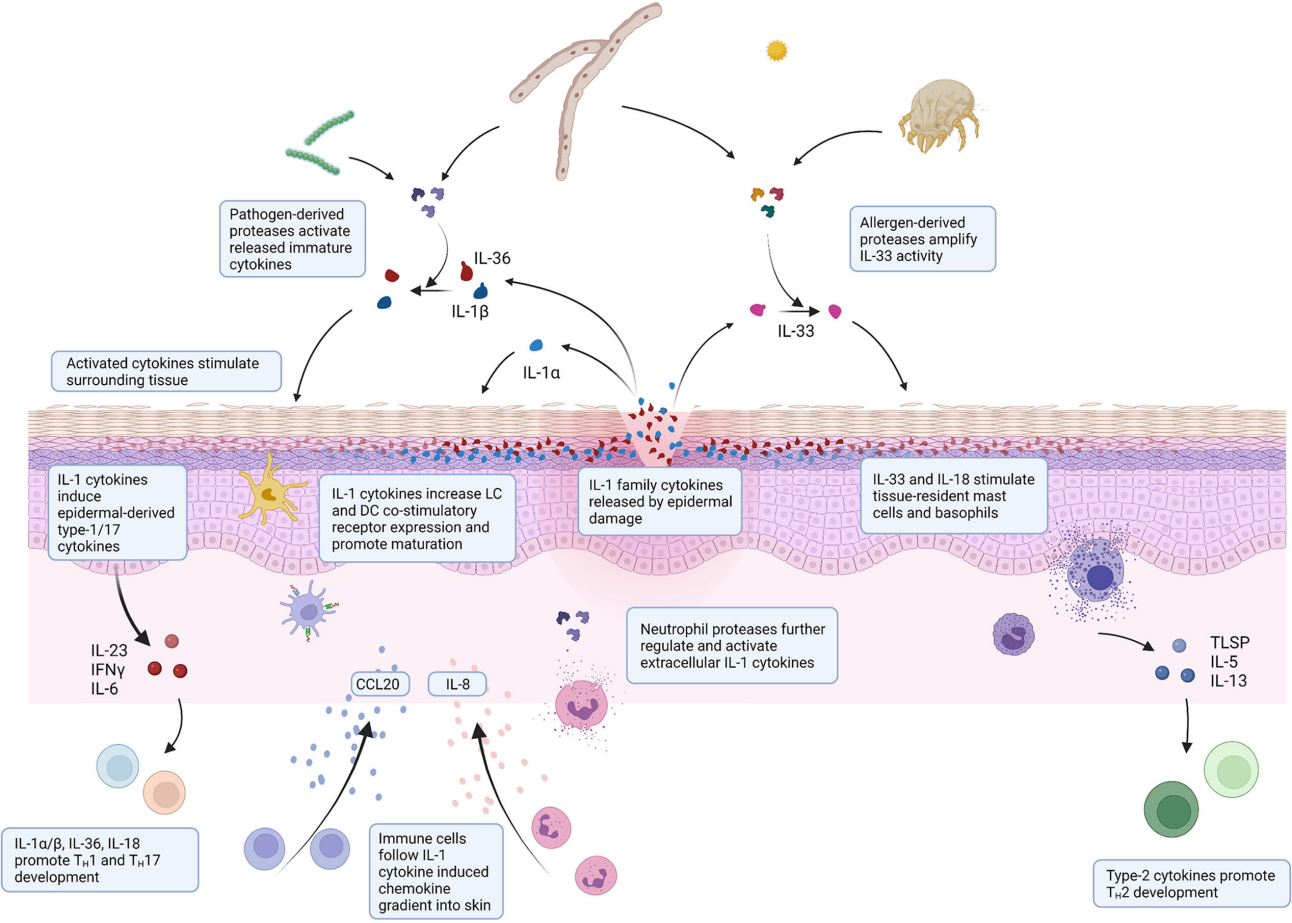
Environmental interaction of IL-1 family cytokines in skin injury. Schematic illustrating the mechanisms by which IL-1 family cytokines can sense environmental threats and orchestrate a subsequent immunological response. Epidermal damage through infection or injury releases IL-1 cytokines. Immature cytokines are activated by environmental proteases expressed by pathogens during infection or allergens present in the environment. Active released cytokines stimulate surrounding epidermis and resident immune cells to facilitate immune cell recruitment to mount an appropriate immune response.

IL-36γ, in particular, is well suited as an epidermal alarmin as it is constitutively expressed in healthy human skin and is found even in the upper layers of the stratum corneum, where it is most likely to come into contact with invading pathogens following injury (14, 16). Its sensitivity to exogenous proteolytic activation and presence in the epidermal layers as a store of inactive inflammatory cytokine means that a small amount of pathogen-derived protease capable of activating IL-36γ can rapidly generate a strong inflammatory signal without relying on protein synthesis. **Figure 2** outlines the mechanism by which IL-1 family cytokines may sense environmental threats and warn the surrounding tissue to potential damage or infection.

Although not all IL-1 family cytokines act with an alarmin function in skin, several still have a crucial role in communicating the presence of environmental threats through their regulation by inflammasomes. Upon recognition of PAMPs and DAMPs by inflammasome sensors such as NLRP3, NLRC4, NAIP and AIM2, inflammasome complexes form and act as platforms for the activation of IL-1β, IL-18 and IL-37 (20). Inflammasome-mediated activation of the pore-forming protein gasdermin D then facilitates release of these cytokines into the surrounding tissue, thereby signalling the presence of an environmental threat (23). Whilst IL-1β must be induced and expressed prior to activation and secretion, IL-18 is constitutively expressed in the epidermis and even secreted in its immature form and is therefore available for activation immediately upon inflammasome formation (24). Furthermore, the inflammasome complexes themselves can be released into the surrounding extracellular tissue following activation which allows for maturation of extracellular IL-18 and IL-1β, amplifying the inflammatory response (25).

### Microbial Interaction

In addition to alerting the skin to potential threats, many aspects of IL-1 family biology also help the immune system discriminate harmful pathogenic microbes from commensals. IL-1β has been shown to have a role facilitating the discrimination of pathogens from commensals in skin through its influence on peripheral tolerance. Colonisation of skin by commensals during early life gives rise to commensal-specific regulatory T cell populations, which provide the host with commensal-specific tolerance and prevent unwanted inflammation (26). Recent evidence in mouse models shows IL-1β can influence which colonising microbes the immune system tolerates by negatively regulating T_reg_ cells (27). During early life colonisation of skin by *S. aureus*, virulence factors and cytotoxic proteins expressed by *S. aureus* that commensals lack induce expression of IL-1β. The induced IL-1β prevents the expansion of *S. aureus*-specific T_reg_ cells, meaning subsequent exposure to *S. aureus* does not result in the expansion of a strong tolerogenic population of T_reg_ cells, thereby allowing an inflammatory response to the skin pathogen (27).

Unlike IL-1β and IL-18, IL-36 cytokines are not activated by caspase-1 following inflammasome activation (3, 28). Whilst the mechanisms of IL-36 cytokine release have not been definitively outlined, cell death induced by pathogen-mediated damage has been demonstrated as one method. Indeed, the literature highlights a trend of IL-1 family cytokines to be released and activated by proteins associated with pathogen virulence. Phenol-soluble modulin (PSM)α virulence peptides secreted by *S. aureus* release IL-1α and IL-36 cytokines from keratinocytes through cellular damage (29, 30). As previously highlighted, the virulence factors SpeB and Alp 1 secreted during infection by *S. pyogenes* and *A. fumigatus* respectively activate IL-36γ, and SpeB has been shown to activate extracellular pro-IL-1β (15, 31). Recently the *Yersinia pestis* virulence factor YopJ, which inhibits NF-kB signalling, has been shown to trigger release of IL-1β and IL-18 *via* caspase-8-dependent gasdermin D activation (32, 33). By requiring either active or pathogen-dependent release from a damaged or activated cell, or activation by a pathogen-derived protease, or a combination of the two, these cytokines help the skin’s immune system discriminate the harmful from the harmless and allow appropriate anti-microbial or wound-healing responses.

IL-33 is an immunological outlier of the IL-1 family of cytokines given it is associated with the paradigmatic Type II diseases rather than with the innate immunopathology driven diseases associated with the other members. It has been shown to contribute to the downregulation of both filaggrin and human beta defensin 2 expression (34, 35). However, IL-33 shares many characteristics with its fellow IL-1 family cytokines in its alarmin function and as a sensor of environmental threats. IL-33 also appears to have a role in detecting cellular stress. Keratinocytes increase expression of IL-33 when experiencing hypo-osmotic stress. The induced IL-33 was observed to localise at the nucleus rather than being secreted, leading the authors to hypothesise that the increased expression acts as a failsafe in circumstances where stress progresses to damage, thus releasing IL-33 and inducing inflammation (36). In addition to the above mentioned IL-33 functions, this may be of relevance in atopic dermatitis (AD) and eczema where a loss of barrier integrity leaves the skin more exposed to external osmotic stress. Indeed, exposure to fresh water is known to exacerbate AD and eczema (37). A similar concept of increasing alarmin expression as a failsafe has also been suggested to occur with IL-1α, whereby the presence of the commensal *Staphylococcus epidermidis* allows the epidermis to maintain a level of intracellular IL-1α, keeping it primed for an efficient response in the event of damage (38). Similar to IL-1 and IL-36 cytokines, IL-33 has been shown to be truncated to potently active forms by numerous exogenous proteases, including pollen- and house dust mite-derived proteases (39). IL-33 is retained in the cell nucleus and cytosol as an intracellular store, is active in its immature form, and is released upon cell damage or programmed cell death (40). Yet, proteolytic truncation can dramatically increase its biological activity and will therefore generate a more potent alarmin once exposed to the external environment. In contrast to the other IL-1 family cytokines, IL-33 drives Type II immune responses, so susceptibility to proteolytic activation by an alternative set of exogenous proteases may function as a method of fine-tuning the inflammatory alarm depending upon the proteolytic environment experienced by the alarmin once released from the cell.

### Pathological Consequences

Whilst IL-1 family cytokines are well suited as sensors of potential threats, their sensitivity to proteolytic activation and interaction with environmental microbes may also lead to the development of pathological inflammation. IL-36γ and *Cutibacterium acnes* have also been implicated in the pathomechanism of drug-induced acneiform skin toxicity (41). Satoh et al. report that EGFR/MEK inhibition in patients colonised with the commensal causes synergistic expression of IL-36γ. *C. acnes* mediated activation of NF-kB combined with increased expression of Krüppel-like factor 4 as a result of EGFRi/MEKi treatment strongly upregulated IL-36γ expression, which in turn lead to inflammatory skin-based toxicity (41). *S. pyogenes* tonsillar infection has a well-established strong aetiological association with guttate psoriasis – an acute eruptive form of psoriasis seen largely in children – and is the strongest environmental factor in triggering and exacerbating psoriatic skin lesions (42, 43). It has therefore been hypothesised that *S. pyogenes*-mediated proteolysis of IL-1β and IL-36γ during infection may trigger inflammation and lead to the development of inflammatory disease. The importance of balanced proteolytic regulation between host and commensal in epidermal homeostasis is underlined in patients with Netherton Syndrome (NS), in which the endogenous protease inhibitor LEKTI-1 is non-functional. Patients with NS suffer predominantly IL-17- and IL-36-driven cutaneous inflammation in conjunction with a *S. aureus* and *S. epidermidis* dominated microbiome (44, 45). A recent study conducted in mice has demonstrated the lack of adequate endogenous protease inhibition imparted by LEKTI-1 promotes dysbiosis, which in turn alters the proteolytic balance of the epidermis through the secretion of proteolytic virulence factors, inducing skin damage and promoting inflammation (45).

The pathological consequence of protease sensitivity is not limited to exogenous proteases as many endogenous proteases are also capable of truncating IL-1 family precursors to biologically active forms. IL-1α can be truncated to a more potent form by numerous proteases including calpain, granzyme B and neutrophil proteases to a more biologically potent form (46). Yet ‘unplanned’ truncation by endogenous proteases often generates less biologically active forms than their intended activators. IL-1β, for example, can be truncated by neutrophil elastase, proteinase-3 and cathepsin G, however the resultant forms are less active than caspase-1-truncated IL-1β (47–49). Nevertheless these mechanisms of activation are implicated in a number of inflammatory conditions. Proteases secreted by neutrophils, which are prominent in psoriasis plaques, have been shown to truncate immature IL-1 family cytokines including IL-1α, IL-1β, and the IL-36 cytokines, thereby potentially amplifying the inflammatory environment once recruited to the skin (50). Cathepsin G, neutrophil elastase and proteinase-3 have been demonstrated to activate IL-36α and IL-36γ (16, 51). There is some debate over the inflammatory impact of neutrophil-derived proteases as they are also involved in activating immature IL-36Ra and may also be involved in proteolytically de-activating IL-1 family cytokines following prolonged exposure (3, 52). Given the abundance of neutrophils and IL-36γ in psoriasis plaques, it seems likely neutrophils will exacerbate IL-36-mediated inflammation in psoriasis, however the contradictions present in the literature demonstrate there is still a lack of understanding on the overall neutrophil-mediated impact on IL-1 and IL-36 mediated inflammation *in vivo*.

In addition to promoting inflammatory disease, proteolysis and microbe-induced expression of IL-1 family members can promote atopic disease. Whilst IL-18 is primarily associated with promoting Type I immune responses, IL-18 secretion is known to be induced by *S. aureus* colonisation in cases of AD (53). This is particularly interesting when considered in conjunction with mast cell digestion allowing activation of extracellular IL-18 in the absence of caspase-1 activity (54). In a mouse model of atopic dermatitis, epicutaneous *S. aureus* has been shown to induce IL-36-dependent signalling and a subsequent increase in IgE and IL-4 production (55). However, as yet there are no data implicating this mechanism is present in humans and there is no comprehensive link between IL-36 signalling and factors driving immunoglobulin class switching.

IL-1α has been demonstrated to be instrumental in the development of chronic inflammation in filaggrin-deficient mice. In these mice, skin injury and dysbiosis drive IL-1α expression and secretion from keratinocytes, which in turn causes chronic inflammation (56). IL-33 has also been implicated in the environmental allergen-mediated triggering of allergic inflammation. Recent evidence shows environmental allergens including house dust mites, *Alternaria alternata*, *Aspergillus fumigatus* and cat dander can initiate intracellular cleavage and subsequent secretion of active IL-33 as a result of RIPK-caspase-8 ripoptosome activation in epithelial cells (57). The released active IL-33 is then free to induce pro-atopic responses from surrounding tissue. Considering the evidence that IL-33 is also susceptible to proteolytic activation by many proteases produced by these environmental allergens when in the extracellular space, it seems likely pathological IL-33-mediated signalling would result from exposure to these allergens.

## Orchestrating Immune Responses

Once released and activated, IL-1 family cytokines help to orchestrate an appropriate immune response through interaction with their local cellular environment (**Figure 2**). IL-1 family cytokines have an impact to some extent on all cells within the skin and elicit a response from neighbouring keratinocytes and infiltrating immune cells. In doing so they influence both the innate and adaptive response.

### Effects on the Keratinocyte Barrier

Within the epidermis, keratinocytes are strong responders to IL-1 family cytokines. Given their abundance in comparison to infiltrating immune cells, they are arguably the most important initial responders to IL-1 family cytokines and act to both fight infection through release of anti-microbial peptides and amplify inflammatory signals through release of a range of chemokines and cytokines. As many IL-1 family cytokines transduce signal through MyD88, keratinocytes respond to pro-inflammatory IL-1 family cytokines in a very similar fashion. Indeed, a recent study examining transcriptional differences in keratinocytes following IL-1β and IL-36 cytokine stimulation found nearly all IL-1β responses were replicated by IL-36 stimulation (58). In response to IL-1α, IL-1β, and IL-36 stimulation, keratinocytes express and release a host of antimicrobial peptides including human beta defensins, LL-37, and S100 proteins which directly combat infection (59, 60). They increase protein levels of many key pro-inflammatory cytokines including IL-23, IL-17C, TNFα, as well as other IL-1 family cytokines that will further amplify an inflammatory response (1, 61). Keratinocytes also secrete chemokines following stimulation with IL-1 family cytokines. IL-1α, IL-1β, and IL-36 cytokines all induce secretion of IL-8 (CXCL8) and CCL2 (aka MCP-1) – strong inducers of neutrophil and myeloid cell chemotaxis respectively – and CCL20 – responsible for the influx of cells expressing CCR6, which include T_H_17, gamma delta (γδ) T cells and Type III innate lymphoid cells (ILC3s) (58, 62). Keratinocytes also secrete CXCL10 in response to IL-18 stimulation; a chemoattractant of CXCR3-expressing cells including CD8 and CD4 T cells (63).

In addition to inducing expression and secretion of cytokines, chemokines and peptides from keratinocytes, IL-1 family cytokines can also induce cellular changes in keratinocytes that influence their barrier function. Recently IL-1β has been shown to promote cell-intrinsic immunity in epithelial cells by inducing activation of IRF3 and therefore driving an innate anti-viral response. IL-1β was found to induce the release of mitochondrial DNA into the cytosol of stimulated cells, subsequently activating the cGAS-STING pathway and activating IRF3 (64). There is evidence to suggest IL-1α stimulation of keratinocytes reduces the adherence of *S. pyogenes*, thereby reducing the likelihood of invading bacteria establishing an infection. This was hypothesised to be a result of changes in their expression of receptors utilised by *S. pyogenes* in adhering to keratinocytes (65). IL-18 stimulation of keratinocytes is known to increase expression of molecules including MHC-II, increasing their capacity to present antigens to infiltrating T cells (63).

### Effects on the Skin’s Immune Compartment

In addition to their effects on the epidermis, the IL-1 family cytokines have a significant effect on the immune compartment. Langherans cells (LCs) and dendritic cells (DCs) reside in the epidermis and dermis, respectively, sampling their environment for threats to respond to. LCs express high levels of IL-36R and IL-1R in comparison to blood-derived myeloid cells and respond to stimulation by secreting pro-inflammatory mediators and expressing co-stimulatory receptors. Whilst traditionally IL-1β has been thought of as a crucial cytokine along with TNFα for the maturation and migration of LCs (66), IL-36β has been shown to be equally as potent as IL-1β in stimulating LCs (67). Dendritic cells, on the other hand, are slightly more varied in their expression and response to IL-1β and IL-36 cytokines. CD3a^+^ DCs express high levels of IL-36R yet have limited expression of IL-1RAcP, and conversely CD14^+^ DCs express high levels of IL-1RAcP but do not express IL-36R. As a result, dermal DCs are not very responsive to IL-36 stimulation but respond well to IL-1β by similarly expressing pro-inflammatory cytokines and co-stimulatory receptors (67).

Neutrophils and macrophages recruited to the skin during inflammation are also influenced by IL-1 family cytokines. IL-1α, IL-1β and IL-18 have been shown to activate neutrophils, increasing oxidative burst and inducing calcium-dependent degranulation (68, 69). Macrophages react variably to IL-1 family cytokines depending on their differentiation. M0 macrophages are responsive to both IL-1β and IL-36 cytokines, but more responsive to IL-1β than IL-36. M1 macrophages respond to IL-1β but not to IL-36, whilst M2 macrophages are equally responsive to both IL-36 cytokines and IL-1β (67). IL-18, originally termed IFN-gamma (IFN-γ) inducing factor, plays a significant role in the activation of mononuclear cells facilitating improved clearance of both intracellular and extracellular bacterial infections as a result of its induction of IFN-y from multiple cellular sources (70).

Innate lymphoid cells (ILCs) both reside within the dermis and epidermis as tissue-resident cells and are actively recruited to the skin during inflammation. IL-1β has recently been shown to facilitate plasticity in ILCs. ILC2s stimulated with IL-1β begin to express low levels of T-bet and IL-12Rβ2, allowing a phenotypic switch to ILC1s following IL-12 stimulation (71). Similarly, ILC2s treated with IL-1β, TGF-b and IL-23 can adopt an ILC3 phenotype by expressing RORγt and producing IL-17 (72). IL-18 in combination with IL-15 has been shown to be important in the proliferation of ILC3s and can contribute to their role in epithelial barrier function by inducing expression of IL-22 (73).

In addition to their effects on cells of the innate immune system, IL-1 family cytokines significantly influence adaptive immunity, both directly and indirectly. IL-1β is well established to potentiate production of IFN-γ and act as a driver of T_H_17 differentiation through direct stimulation (74). Recently, IL-1β has also been shown to promote T_H_17 differentiation indirectly *via* activation of DCs. IL-1β-primed DCs increased expression of CD14 and induced RORγt expression and IL-17 production in CD4 memory T cells in a CD14 dependent manner (75). Whilst murine T cells are well established as expressing IL-36R and are directly responsive to IL-36 stimulation, the sensitivity of human T cells to IL-36 cytokines is less clear. Early research could not demonstrate a direct effect of IL-36 stimulation on human T cells, however a later report by Penha et al. show expression of IL-36R in blood- and intestinal-derived T cells and dose-dependent CD4 T cell expansion following stimulation with IL-36β (62, 67, 76). IL-36 cytokines have also similarly been shown to promote CD4 T cell proliferation through their activation of DCs, other antigen presenting cells, and the induction of polarising cytokines (62, 77, 78). Interestingly, in mice IL-36γ has been shown to prevent the development of T_reg_ cells, instead promoting development of T_H_9 effector cells (79).

Unlike the other IL-1 family members, IL-33’s influence on the immune compartment drives Type II immunity and is strongly linked with promoting T_H_2-driven diseases such as AD. IL-33 has been shown to induce the expression of CCL17 from AD-derived keratinocytes; a chemokine responsible for attracting CCR4 positive cells associated with Type II immune responses (80). Mast cells undergo activation and maturation following IL-33 stimulation and become more responsive to IgE and IgG. These cells also respond to both IL-18 and IL-33 by producing Type II polarising cytokines such as TSLP, IL-4, and IL-13 (81–83) and therefore promote T_H_2 development. T_H_2 cells themselves are also known to express ST2 and respond directly to IL-33 stimulation by producing IL-5 and IL-13 (84, 85).

In addition to driving T_H_2 development, IL-33 has been well described as a driver of ILC2 proliferation (86). ILC2s are thought to play a prominent role in the pathogenesis of AD. Their numbers are found at increased levels in the skin of AD patients when compared to that of psoriasis patients (87). Indeed mouse models of AD induced by transgenic IL-33 expression can be effectively treated by the specific depletion of ILC2s (88). Despite their role in promoting pathological Type II inflammation, IL-33 and ILC2s are also associated with protection. Numerous publications demonstrate their importance for protection against gut tissue damage and gastro-intestinal infections (89–91), and similarly has been shown as promoting ILC2-mediated wound healing in skin (92).

## Resolving and Regulating IL-1 Family Inflammation

All IL-1 family members are tightly controlled (93) on multiple levels including RNA stability, translation, secretion and activation by proteases as well as soluble receptors, receptor antagonists and binding proteins (**Table 1**). This significant level of control may also illustrate the powerful pro-inflammatory properties of IL-1 family members and disturbance in any of these mechanisms can lead to auto-inflammatory (sometimes clinically dramatic) disease manifestations.

**Table 1:** Table showing the regulatory mechanisms controlling IL-1 family mediated signalling.

IL-1 family member	Antagonist	Other inflammation limiting mechanisms
**IL-1α (signal transduction by receptor complex IL-1R1+IL-1R3)**	IL-1Ra, IL-1R2, soluble receptors (sIL-1R1 & sIL-1R2), IL-37/SIGIRR	RNA stability, protein secretion, sheddase activity; miRNA (94)
**IL-1β**	IL-1Ra, IL-1R2, soluble receptors, IL-37/SIGIRR	RNA stability, caspase activation, protein secretion, sheddase activity; reduced inflammasome activity/caspase 1
	*Note: the affinity of sIL-1R2 for IL-1β is increased by the formation of trimeric soluble complexes with sIL-1R3 (* 95)	
**IL-36 agonists**	IL-36Ra	Protease availability; activation of IL-36Ra by elastase
	IL-37	
	IL-38	
**IL-18**	IL-18BP, IL-37	
**IL-33**	sST2	Caspase-3/-7 mediated inactivation, oxidation, nuclear sequestration
**IL-37**		Enhanced IL-18BP activity
		Binds to IL-18R1 and SIGIRR
**IL-38**		Uncharacterised mechanism

IL-1Ra, IL-1 receptor antagonist; BP, binding protein.

There is limited knowledge about how counter-regulatory IL-1 family mechanisms are activated and regulated. While counter-regulatory activity happens on the level of receptor expression, soluble receptor release, signalling molecule stability/ubiquitination, translocation, activation and mRNA stability, here we highlight protein antagonists for each IL-1 family member. The cutaneous dynamics of all counter-regulatory mechanisms have not been well investigated and as such no complete picture is available for the IL-1 family relevant resolution phase of inflammatory epithelial responses *in vivo*. For blood derived monocytes, persistent versus resolving inflammation experimental setups have been investigated (96) and showed that IL-1β, IL-1Ra and soluble receptors are all released between 4 and 14h during the course of both resolving and persistent inflammation *in vitro*.

IL-1Ra is the first described endogenous receptor antagonist. Different variants, including an intracellular form lacking a leader sequence (97), have been described which add to its complex biology. In the skin, IL-1Ra is produced abundantly by keratinocytes, infiltrating neutrophils and infiltrating or resident antigen presenting cells. IL-1Ra binds to the IL-1R1 but fails to induce recruitment of the co-receptor IL-1R3 (aka IL-1RAcP), thus preventing signal transduction *via* the IL-1R. This is how it works as a competitive antagonist for both IL-1α and IL-1β. IL-1R type 2 (CD121b) is a decoy receptor which binds to IL-1α and IL-1β with high affinity and IL-1RA with lower affinity. While IL-1R2 interacts with IL-1R3 this interaction does not result in signal transduction. In addition to its membrane bound form IL-1R2 has also been found as a soluble protein either as a result of shedding or alternative splicing and has been shown to be active in the cytosol binding to IL-1α and preventing its cleavage and activation. Similarly, soluble receptors have been described for IL-33, sequestering extracellular IL-33 and limiting subsequent signalling (98). All IL-1α/β relevant molecules mentioned, including the IL-1RA and type 2 IL-1R are expressed by keratinocytes (99). The IL-36 cytokines are also regulated through a receptor antagonist, IL-36Ra. IL-36Ra functions in an analogous manner to IL-1Ra, binding its cognate receptor IL-36R without recruiting the accessory protein IL-1RAcP, thus preventing signal transduction.

IL-38 is another anti-inflammatory cytokine belonging to the IL-36 sub-family. Whilst this cytokine is thought to be an important regulator of IL-36 dependent inflammation and has been shown to be a general regulator of IL-1-family mediated inflammation, its precise mechanism of action is not wholly understood. There is evidence to suggest it may directly antagonise IL-36R like IL-36Ra, however this has not yet been conclusively demonstrated. Nevertheless, it has been shown to inhibit IL-36γ-induced NF-kB activity in keratinocytes and seems to be important in regulating inflammation in psoriasis. Its expression is lower in psoriatic skin than in healthy skin and increases following anti-IL-17A treatment in a manner that positively correlates with therapeutic efficacy (100).

While those cells producing IL-1α/β also harbour the full repertoire of antagonistic molecules the same is not necessarily true for the IL-18 system. Here keratinocytes are recognised producers of IL-18 as of relevance in psoriasis, cutaneous lupus and chronic eczema, however a main source of IL-18BP in the skin organ are fibroblasts (101) which are not known to secrete IL-18. Yet it has recently been shown that the cleaved pro-peptide from IL-18 can interact with mature IL-18, potentially abrogating its ability to engage with its receptor, highlighting an innate method of limiting IL-18 signalling without requiring expression of IL-18BP (102).

It is of interest that another anti-inflammatory molecule impacting on IL-1 family members, IL-37 (103), uses the IL-18R in addition to SIGIRR (IL-1R8) for its downstream effects (104). IL-1R8 interacts with IRAK and TRAF6 thus competing with IL-1 or TLR activated signalling. As previously mentioned, Gudjonsson et al. have clearly shown that the signalling events induced by IL-36 are mostly identical to IL-1 induced signalling (58). The difference in their pathophysiology relies on the compartment where they are expressed and events leading to cleavage of pro-forms into bioactive molecules. Regarding IL-1R8, it can attenuate inflammatory responses induced by IL-1, IL-18, IL-33, IL-36, and TLR. A recent study in *SIGIRR^-/-^* mice demonstrates its importance in regulating IL-36-mediated inflammation (105). The anti-inflammatory molecule IL-37 can also bind to IL-18BP and seems to enhance its effects. IL-37 along with all IL-36 members and the IL-36 receptor antagonist IL-36Ra are all expressed by human keratinocytes.

Studies examining the regulation of IL-33 have revealed some interesting mechanisms of limiting IL-1 cytokine activity. Whilst proteolytic regulation of the IL-1 family cytokines is often associated with their activation, it has been demonstrated that IL-33, already active in its native form, is inactivated following truncation by caspase-3 and caspase-7 during apoptosis (106, 107). Interestingly, this inhibition is mediated by truncation of a specific amino acid sequence that is absent in all other IL-1 family cytokines, suggesting specific regulation of IL-33 during apoptosis (106). Intriguingly IL-33 has recently been demonstrated to exhibit an intrinsic method of limiting prolonged activity. IL-33 quickly loses its biological function following release into the extracellular environment as oxidation of critical cysteine residues in IL-33 causes a conformational switch to an inactive form (108). As other IL-1 family cytokines also contain free cysteine residues it is possible other IL-1 family members are regulated in a similar manner. Indeed Cohen et al. demonstrate IL-1α, IL-18, IL-36α and IL-36γ all exhibit similar oxidative changes when exposed to an oxidative environment (108).

Within the skin compartment much inflammatory activity related to the IL-1 family is based in the epidermis. As mentioned, dermal resident cells such as fibroblasts are less able to produce IL-1 family agonists but contribute to antagonist secretion including IL-18BP and IL-1Ra. Thus, infiltrating macrophages aside, the overall property of the dermal versus epidermal compartment is more inflammation limiting than pro-inflammatory with regard to IL-1 family molecules.

**Figure 3:**
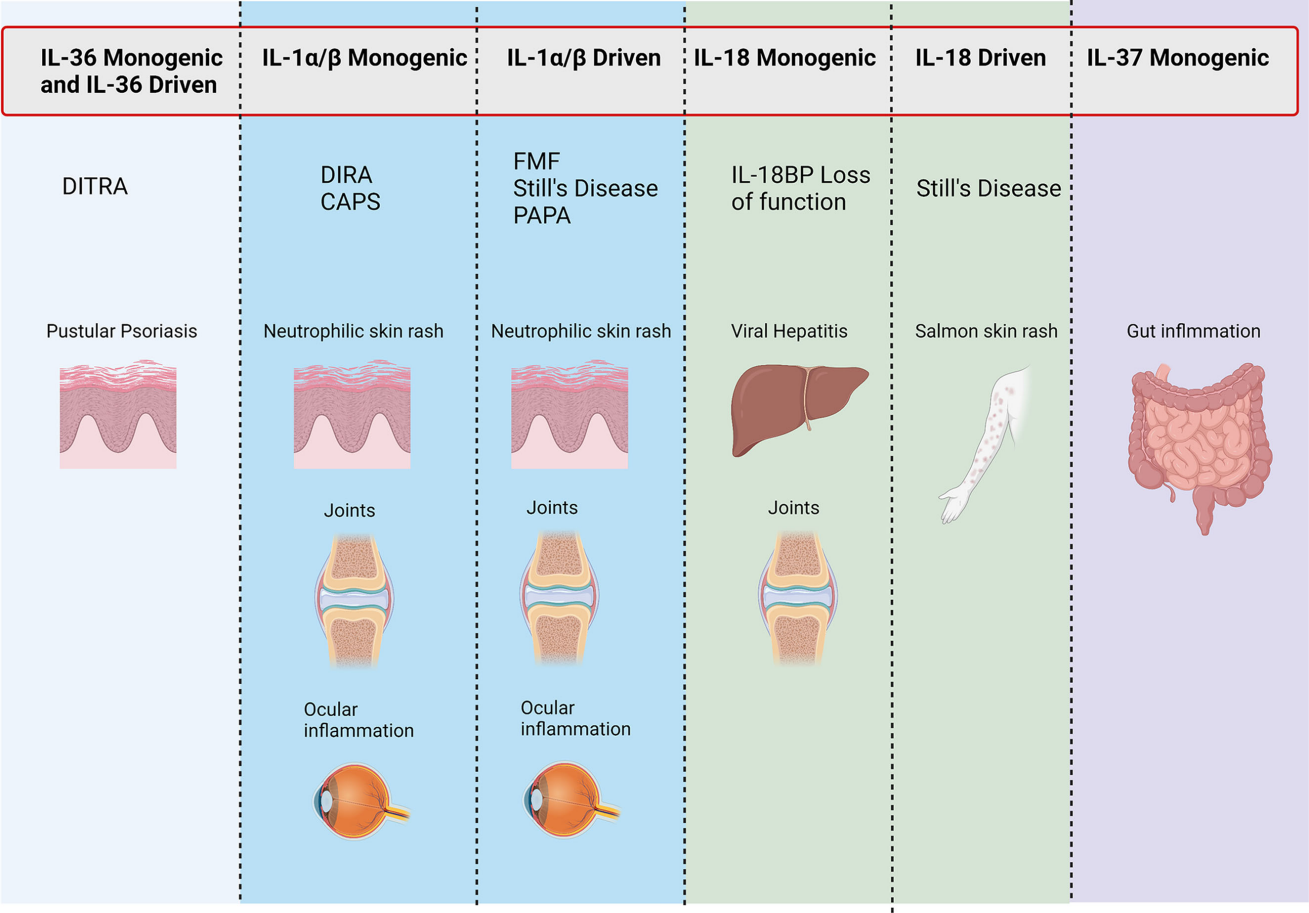
Conditions associated with IL-1 family genetic mutations. Figure outlining symptoms associated with disease-causing mutations in monogenic and IL-1 family driven conditions.

**Figure 4:**
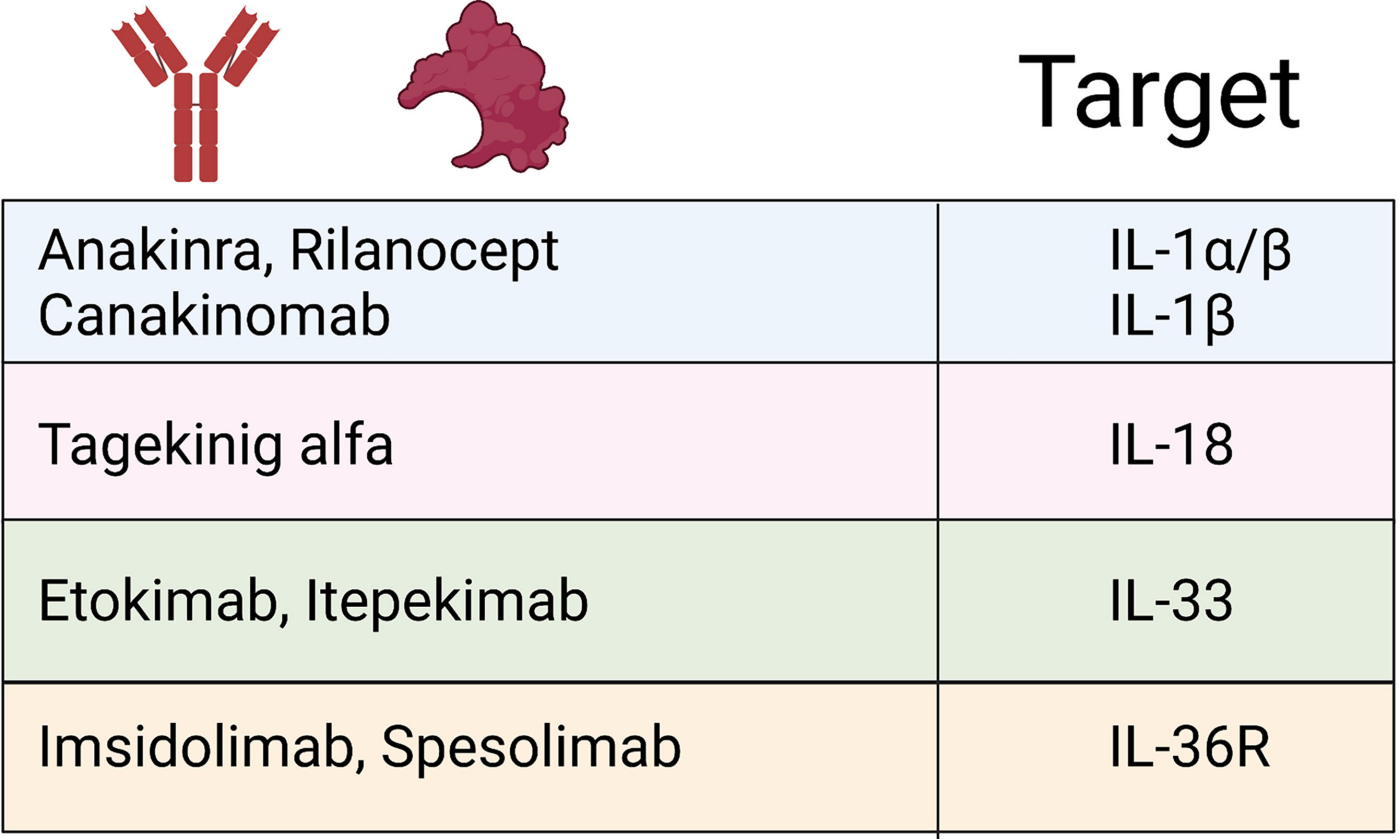
IL-1 family biologics. Schematic outlining current FDA-approved biologics targeting IL-1 family cytokines and signalling pathways that have demonstrated efficacy treating IL-1 cytokine-driven conditions.

The importance of both adequate regulation and resolution of inflammation by the IL-1 family cytokines is most clearly demonstrated when examining monogenic mutations that directly affect the regulation of IL-1 family cytokines (summarized in **Figure 3**). This is most striking in conditions that result from the loss of the IL-1 family antagonists. Deficiency of the IL-1 Receptor Antagonist **(DIRA)** is an autosomal recessive disease caused by mutations of *IL1RN* that results in non-expression of the IL-1 receptor antagonist and subsequent unopposed IL-1 receptor activation by IL-1α and IL-1β (109). DIRA is most commonly associated with a pustular rash, joint swelling and periostitis. DIRA has been successfully treated with the anti-IL-1 biologics Anakinra (recombinant IL-1Ra) (110, 111), Canakinumab (112) (IL-1β blocking antibody) and Rilonacept (fusion protein of IL-1 receptor and IgG Fc) (**Figure 4**) (113).

Similarly, loss of function mutations in *IL36RN*, which encodes IL-36Ra, leads to the development of DITRA (Deficiency of interleukin-36 receptor antagonist) in which unchecked IL-36 signalling causes significant systemic inflammation (114). In addition to the immediate inflammatory consequence of enhanced IL-36 signalling, the lack of IL-36-specific inhibition has been shown to promote extensive antigen-dependent expansion of T_H_17 cells and subsequent production of IL-17A, promoting T cell-mediated inflammation (115). Affected patients develop GPP psoriasis with a systemic inflammation and fever. GPP has successfully been treated with anti-IL-36R therapy (**Figure 4**) (116). If left untreated, both DIRA and DITRA can prove fatal, demonstrating the importance of proper regulation of IL-1 and IL-36 signalling (117, 118).

Numerous genetic defects that occur in the inflammasome-activation pathway demonstrate the importance of properly regulated inflammasome activation. Examples of both gain of function mutations in inflammasome activation and loss of function mutations in inflammasome regulation have been well reported. Cryopyrin-Associated Periodic Syndrome (CAPS) represents autosomal dominant gain of function mutations that results in over activation of the inflammasome and increased conversion of pro-caspase-1 to activate caspase-1 and this results in increased IL-1β and IL-18 protein levels (119, 120). These conditions include Familial Cold Autoinflammatory Syndrome (FCAS) and Muckle-Wells Syndrome (MWS) caused by mutations in the CIAS1 gene (NLRP3) which encodes cryopyrin (121–123). CINCA (chronic infantile neurologic, cutaneous and arthritis syndrome) was later revealed to be caused by a spontaneous mutation in the same gene and called, Neonatal Onset Multisystem Inflammatory Disease (NOMID) (124, 125). Recent publications have also identified gain of function mutations in *NLRC4* resulting in similar aberrant activation of the NLRC4 inflammasome (126). Amongst severe systemic symptoms such as macrophage activation syndrome and periodic fever, patients also exhibit severe urticarial rashes that are marked by significant neutrophil infiltrates. Interestingly, patients with NLRC4 mutations exhibit higher levels of IL-1β and IL-18 than those with NLRP3 mutations, and have been shown to respond well to IL-18BP treatment (127–129). CAPS patients respond well to IL-1 blockade, with Anakinra, Rilonacept and Canakinumab all showing efficacy (130–135).

FMF (Familial Mediterranean Fever) results from a breakdown in regulation of inflammasome activity caused by mutations in the MEFV gene which encodes pyrin (136, 137). The precise role of pyrin in the pathogenesis of FMF is heavily debated. Pyrin interacts with ASC, a component of the inflammasome and sequesters ASC, making unable to form an inflammasome (138, 139). Wildtype pyrin has also been demonstrated to bind to caspase-1 and inhibit IL-1β secretion (138, 140). FMF is commonly treated with colchicine, however, there are reports of IL-1 blockade being successful in the form of Anakinra and Canakinumab (141, 142).

Whilst not common, there are also examples of IL-37 and IL-18BP loss of function giving rise to inflammatory disease. An infant with non-functional IL-37 has been identified to suffer inflammatory bowel disease, however, as yet shows no sign of skin manifestations. The loss of functional IL-18BP resulted in a case of fulminant viral hepatitis and the death of the patient (143, 144).

The IL-1 family cytokines are also well known to have central roles in complex multifactorial inflammatory conditions such as psoriasis, ichthyosis conditions, hidradenitis suppurativa (HS) and AD. As these topics have been extensively reviewed elsewhere (93, 145, 146) this review shall highlight recent examples of inflammatory conditions involving IL-1 family cytokines.

Systemic-onset juvenile idiopathic arthritis (sJIA) and adult-onset Still’s disease (AoSD) are rare systemic auto-inflammatory polygenic disorders, which are accompanied by a broad spectrum of manifestations. The diseases, considered part of a spectrum continuum, are associated with fever, arthritis and skin rashes that are salmon pink in appearance. Skin biopsies show neutrophilic inflammation (147). Both diseases considered can significantly overlap with macrophage activation syndrome (MAS) and feature a cytokine storm. The causes of these entities and immunogenetics are complex and are discussed elsewhere (148). However, a central mechanism appears to be the dysregulated production of IFN-y, IL-1, IL-6 and IL-18, the latter driving IFN-y (148). Targeting of IL-1α/β (Anakinra (149) or Canakinumab (150), as well as recombinant IL-18BP (151) (tadekinig alfa) has shown some success. A hair follicle disease hidradenitis suppurativa (HS), which is dominated by pathological neutrophils, has recently been shown to significantly involve the IL-1 and IL-36 pathways in driving its proinflammatory pathways (152, 153) IL-1β-driven transcription present in HS lesions was found to induce immune cell infiltration to lesions and extracellular matrix degradation (152). IL-36 cytokines were also found to be significantly upregulated, and their regulatory counterparts downregulated, in lesional HS (153).

## IL-1 Family Cytokines in the Promotion of Wound Healing

Wound healing is essential in maintaining the skin integrity and barrier function. It is a complex process, defined by four consecutive but overlapping stages, starting with haemostasis, followed by inflammation, proliferation, and finally tissue remodelling (154, 155). Imbalance in any of these stages can lead to delayed or impaired wound healing. IL-1 family members are known to play a key role in the wound healing process. IL-1α and IL-1β levels are immediately increased following tissue injury, reaching their peak in the first 12 – 24h, underscoring their alarmin function, and return to normal once the proliferation stage of wound healing is complete (156). Interestingly, reinforcing the importance of proteolytic regulation of IL-1 family cytokines, thrombin has recently been demonstrated to truncate IL-1α to a more potent form following its activation in the coagulation cascade (157). Indeed, a mouse model expressing IL-1α that cannot be truncated by thrombin exhibits delayed wound closure (157).

As already alluded to, IL-1 cytokines promote the initial neutrophil recruitment to the site of injury, facilitating the removal of debris and the prevention of a potential bacterial infection (158). Similarly to TNFα, both IL-1α and IL-1β facilitate wound healing by inducing fibroblast and keratinocyte proliferation, the production and degradation of extracellular matrix (ECM) proteins (collagens, elastins, fibronectins and laminins), fibroblast chemotaxis, and by exerting immune modulatory functions (156). The majority of these effects are obtained *via* the production of various growth factors by IL-1 stimulated cells. *In vitro* experiments showed that IL-1 produced by human keratinocytes stimulate the production of transforming growth factor alpha (TGF-α) in an autocrine fashion (159). TGF-α increases keratinocyte migration (160) and proliferation (161–163) as well as their expression of K6 and K16, markers of a hyperproliferative state (164). Furthermore, keratinocyte derived IL-1 stimulates fibroblasts to produce other growth factors with important roles in keratinocyte proliferation, including keratinocyte growth factor (KGF) (165–167) and granulocyte–macrophage colony-stimulating factor (GM-CSF), creating a double paracrine loop (168, 169). In addition, IL-1 indirectly induces dermal fibroblast and arterial smooth muscle cell proliferation by stimulating their production of platelet-derived growth factor (PDGF) AA (170). The newly forming tissue during wound repair has a high oxygen demand, therefore, neovascularisation is an essential part of normal wound healing. IL-1β has been shown to induce vascular endothelial growth factor (VEGF) production by keratinocytes, a key factor in neovascularisation, although to a lesser extent compared to TNFα (171, 172). Interestingly, the bacterial microbiome has been shown to be important in promoting wound healing by inducing IL-1α and IL-1β expression during wounding. A recent study has shown a low bacterial quantity and diversity in mice skin correlates with poor wound-induced hair follicle neogenesis (WIHN), and that increasing bacterial counts, even pathological *S. aureus*, improves WIHN (173). This microbiome-induced promotion of skin regeneration was shown to be dependent on keratinocyte-specific IL-1β signalling. Furthermore, in a small human trial the use of topical antibiotics prior to wounding was demonstrated to prolong the healing process (173).

The various growth factors resulting from the keratinocyte-fibroblast crosstalk are essential in the proliferation of the epithelial stem cell pool (174). Epithelial stem cell mobilisation is crucial in the proliferative phase of wound healing. Progenitor cells derived from interfollicular epidermal and hair follicle stem cells are mobilised to rapidly restore skin integrity (175, 176). However, the signals that trigger the activation of these stem cells proximal to the wound are not fully understood. Recent work conducted in mice indicates the synergistic involvement of IL-1α and IL-7, secreted by keratinocytes, in the expansion of epidermal γδ T cells (177). γδ T cells are resident epidermal cells, which upon activation secrete KGF and induce the proliferation of epidermal stem cells (178, 179). Moreover, it has been shown that in mice IL-1β is indispensable in the recall of inflammatory memory of epidermal stem cells, facilitating wound healing following repeated insults (180).

Interestingly, IL-1β and IL-18 have recently been implicated in the activation of cutaneous mucosal associated invariant T (MAIT) cells following their interaction with local microbiota, which results in the promotion of wound healing. Following interaction with *S. epidermidis* in human epidermis, cutaneous MAIT cells have been shown to expand, produce IL-17A and exhibit a transcriptional profile consistent with leukocyte activation and tissue repair in a manner dependent on IL-1 and IL-18 signalling (181). Local IL-1α signalling was shown to be crucial for MAIT cell IL-17A production, whilst IL-18 was required for their expansion. As previously mentioned, the presence of *S. epidermidis* allows the epidermis to maintain a level of IL-1α expression, thus is primed for allowing IL-1 signalling following tissue damage, promoting MAIT cell activation and subsequent tissue repair. IL-1 family cytokines have also been implicated in promoting wound healing through their impact on commensal-specific tissue resident T cells (T_RM_). A recent study identified *S. epidermidis*-specific CD8^+^ T_C_17 cells to co-express GATA3 and constitutively express a Type II transcriptome, including *IL5* and *IL13*. Despite their expression of the Type II transcriptome, protein synthesis was only licenced following stimulation by tissue damage-associated alarmins (182). Stimulation of *S. epidermidis*-specific T_C_17 cells with IL-1α, IL-33 and IL-18 in conjunction with T cell receptor activation induced production of IL-13, which has been shown to promote wound healing (183). Indeed, the authors demonstrated these Tc17 cells were able to recover *S. epidermidis*-accelerated wound healing in IL-13 deficient mice (182).

Less is known on the role of other IL-1 family members in wound healing apart for an important role for IL-33 that is upregulated following cutaneous wounding in mice, peaking 5 days post-injury (92). Unlike IL-1β, IL-33 promotes reepithelization by facilitating the shift from a pro-inflammatory to an anti-inflammatory macrophage profile and stimulates ECM production in both normal (184) and diabetic mice (185). Furthermore, it enhances neoangiogenesis *via* the production of VEGF and von Willebrand factor (185). IL-33-sensitive ILC2 cells have also been shown to play a role in the wound healing process. Rak et al. identified an increase in ST2^+^ ILC2 numbers at sites of healing wounds in both human and mouse cutaneous tissue. The authors demonstrated that IL-33 knockout mice exhibited significantly smaller numbers of activated ILC2s at wound sites and were significantly slower in closing wounds (92). Oshio et al. also demonstrate IL-33 knockout mice exhibit slower wound closure, and identify the importance of nuclear IL-33 in limiting excessive NF-kB-mediated inflammation (186). IL-36 cytokines have also been implicated in wound healing as a result of injury. Jiang et al. demonstrate that release of TLR3 agonists following tissue damage induces expression of IL-36γ, which in turn induces expression of REG3A in keratinocytes to promote re-epithelialization and wound healing (187).

A wound healing response does not always have a beneficial outcome. Tissue trauma and the subsequent wound healing response can contribute to resurgence of dormant tumours under certain circumstances. The production of growth factors, increase in inflammatory mediators and promotion of re-epithelialisation and proliferation have all been implicated in promoting metastasis and tumour dormancy escape (188). Furthermore, inflammation induced in wound healing does not always resolve completely, and can in some cases lead to the development of chronic wounds (189). Indeed, whilst IL-1 family cytokines are important in the wound healing process, their dysregulation can contribute to pathological outcomes. IL-1 family members have been found to play roles in such inflammatory override, with an essential role being attributed to IL-1β (190). It has been shown that non-microbial danger signals resulting from hyperglycaemia and obesity can lead to inflammasome activation and release of IL-1β and IL-18 from a number of different cell types, including monocytes/macrophages, known to be involved in all stages of wound healing (191–196). IL-1β then sustains the inflammasome activity of macrophages, creating a positive feedback loop (197). Moreover, a recent study in diabetic mice showed that beside maintaining an inflammatory macrophage profile, increased levels of IL-1β also result in the persistence of inflammatory cells at the wound site, senescence of fibroblasts and high levels of matrix metalloproteinases (190), which in turn degrade growth factors and extracellular matrix proteins with an important role in wound healing.

Murine models showed that topical inhibition of caspase 1, the inflammasome, IL-1β or its signalling through IL-1R1, results in the switch from a sustained pro-inflammatory macrophage stage to an anti-inflammatory macrophage profile, reduced levels of proinflammatory cytokines (IL-1β, TNFα, IL-6, CXCL1), chemokines (the murine homologue of IL-8, MIP-1α), and matrix metalloproteases, but increased the levels of anti-inflammatory cytokines (TGF-β1, IL-4, IL-10), tissue inhibitor of metallopeptidase-1 (TIMP-1) and growth factors (FGF-2, PDGF-BB, VEGF-A), facilitating effective wound healing (190, 197–200). Treatment with IL-1RA has been also shown to reduce postoperative wound pain due to the reduced inflammatory response (200). These studies highlight IL-1β as the biggest culprit in the maintenance of chronic, non-healing wounds in diabetic patients, suggesting the inflammasome as a potential therapeutic target in the treatment of non-healing diabetic wounds.

Whilst IL-36 signalling has been shown to have some beneficial roles in the wound healing process, excessive signalling also seems detrimental. Studies performed in mice lacking expression of IL-36Ra demonstrated these mice experienced a 3-day delay in wound closure as a result. Similar to IL-1β, the delay in wound closure was associated with an increased influx of inflammatory cells such as neutrophils and macrophages and increased expression of inflammatory cytokines. Interestingly, normal wound healing was restored in this mouse model by inhibition of TLR4, which the authors attributed to the inhibition of hyaluronic acid-mediated activation of TLR4 and subsequent induction of IL-36 signalling (201).

## Concluding Remarks

This review has provided an overview of the role IL-1 family cytokines play in maintaining the skin’s immunological barrier emphasising recent findings that contribute to our understanding. As has been outlined above, IL-1 family cytokines significantly impact on all stages of an immune response in skin, from sensing threats and alerting surrounding tissue to danger, to facilitating the closure of a wound and restoring the skin’s barrier function. However, IL-1 family cytokines are potent cytokines that require tight regulation and balanced signalling. As has been highlighted, a breakdown in adequate regulation often contributes to, if not causes, disease. The dynamic nature of their regulation, particularly at the post-translational level *via* proteolysis, makes understanding their inflammatory outcome in a given biological system difficult. Yet, given the significant impact they have on almost every aspect of immune function in skin, it is important to consider.

## Author Contributions

TM prepared and wrote the manuscript. AB, CB, and MW contributed to the content of the review. MS, DM and MW directed focus. All authors listed contributed intellectually and approved the manuscript for publication.

## Conflict of Interest

The authors declare that the research was conducted in the absence of any commercial or financial relationships that could be construed as a potential conflict of interest.

## Publisher’s Note

All claims expressed in this article are solely those of the authors and do not necessarily represent those of their affiliated organizations, or those of the publisher, the editors and the reviewers. Any product that may be evaluated in this article, or claim that may be made by its manufacturer, is not guaranteed or endorsed by the publisher.
